# Supplementary material for: Regional variation in health is predominantly driven by lifestyle rather than genetics
Source: Nat Commun. 2017 Oct 6;8:801. doi: 10.1038/s41467-017-00497-5 (PMC5630587; doi:10.1038/s41467-017-00497-5)
Supplement: Supplementary file 1 — Supplementary Information [file 41467_2017_497_MOESM1_ESM.pdf]

Description of Supplementary Files

File Name: Supplementary Information
Description: Supplementary Figures, Supplementary Tables and Supplementary Note

File Name: Peer Review File

Supplementary Table 1a. Description of the phenotypes used in the study.

	Trait	Abv.	Description	Units	Log Transformed?	References
anthropometric	Height	Height	Body Height	cm	No	
	Weight	Weight	Body Weight	kg	Yes	
	Bio-impedance Analysis Fat	BIA Fat	% of Fat (Tanita scales)	%	No	
	Waist	Waist	Waist Circumference	cm	Yes	
	Hips	Hips	Hip Circumference	cm	Yes	
	Waist to Hip Ratio	WHR	Waist/Hips	(None)	Yes	
	Body Mass Index	BMI	$Weight/Height^2$	$kg*cm^{-2}$	Yes	
	A Body Shape Index	ABSI	$Waist/(BMI^{2/3}*Height^{1/2})$	$m^{11/6}*kg^{-2/3}$	Yes	Krakauer & Krakauer (2012). Plos One 7, e39504.
metabolic	Creatinine	Creat	Creatinine level in serum	$\mu mol*l^{-1}$	Yes	
	Total Cholesterol	TC	Total Cholesterol level in serum	$\mu mol*l^{-1}$	Yes	
	HDL	HDL	HDL Cholesterol level in serum	$\mu mol*l^{-1}$	Yes	

Supplementary Table 1b. Correlation between all the phenotypes used in the study.

	Creat	TC	HDL	Height	BIA Fat	Weight	Waist	Hips	BMI	ABSI	WHR
Creat	1.00	0.00	-0.21	0.43	-0.35	0.32	0.27	0.08	0.10	0.22	0.32
TC		1.00	0.21	-0.14	0.22	0.05	0.14	0.08	0.15	0.10	0.13
HDL			1.00	-0.29	0.05	-0.44	-0.39	-0.23	-0.32	-0.18	-0.37
Height				1.00	-0.50	0.52	0.28	0.12	-0.04	0.24	0.30
BIA Fat					1.00	0.25	0.35	0.55	0.62	-0.06	0.02
Weight						1.00	0.86	0.75	0.83	0.24	0.59
Waist							1.00	0.75	0.83	0.63	0.80
Hips								1.00	0.80	0.21	0.20
BMI									1.00	0.13	0.49
ABSI										1.00	0.74
WHR											1.00

Supplementary Table 2a. Description of the covariates used in the study.

Covariate group	Covariate	Abv.	Type	Description
base	Sex	Sex	Discrete	Sex of the individual (M: male, F: female)
	Age	Age	Continuous	Age of the individual at clinic appointment
	Clinic	Clinic	Discrete	Clinic where the measurements were taken (9 values)
socioeconomic	SIMD	SIMD	Continuous	Scottish index of multiple deprivation: A deprivation ranking based on living area and calculated using seven domains: income, employment, crime, education, health, housing and geographical access
	Years of education	YE	Continuous	Number of years attending to school/study fulltime
	Household size	HHS	Continuous	Number of people living in the current household
	VehicleRatio	VR	Continuous	Number of vehicles in the household / Household size
	Job status	Job	Discrete	Jobless status (1: currently unemployed, 0: currently working)
lifestyle	Alcohol units	Alcohol	Continuous	Units of alcohol taken in a week
	Smoking status	Smok	Discrete	Smoking history (1: currenly smoking, 0: currently non-smoker)
	Activity level	Activ	Discrete	Level of activity (1: active, 0: non-active)
	FruitDayUnits	FruitU	Continuous	Number of pieces of fruit eat per day
	Fruit Consumption	FruitC	Discrete	Consumption of fruit (1: usual, 0: non-usual)
	Vegetables Consumption	VegC	Discrete	Consumption of vegetables (1: usual, 0: non-usual)
	Fish Consumption	FishC	Discrete	Consumption of fish (1: usual, 0: non-usual)
	Poultry Consumption	PoultC	Discrete	Consumption of poultry (1: usual, 0: non-usual)
	Meat Consumption	MeatC	Discrete	Consumption of meat (1: usual, 0: non-usual)
	Eggs Consumption	EggC	Discrete	Consumption of eggs (1: usual, 0: non-usual)
	Dairy Consumption	DairyC	Discrete	Consumption of dairy (1: usual, 0: non-usual)
genetic	Geographical Principal Components	gPC	Continuous	Variables capturing the genetic stratification in the sample

Supplementary Table 2b. Correlations between socioeconomic and lifestyle covariates used in the study.

	SIMD	YE	HHS	VR	Alcohol	FruitU	FruitC	VegC	FishC	PoultC	MeatC	EggC	DairyC	Smok	Activ
SIMD	1.00	0.17	0.11	0.17	0.02	0.07	0.12	0.10	-0.01	0.01	0.00	0.00	0.02	-0.18	-0.02
YE		1.00	0.06	0.02	0.03	0.08	0.07	0.09	0.00	-0.04	0.04	-0.02	0.02	-0.09	-0.05
HHS			1.00	-0.30	0.00	-0.03	-0.01	0.01	0.01	0.12	0.10	-0.01	0.02	-0.03	0.02
VR				1.00	0.02	0.04	0.05	0.06	-0.01	-0.02	-0.02	-0.01	-0.01	-0.09	0.01
Alcohol					1.00	-0.12	-0.09	-0.01	0.01	0.03	0.13	0.04	0.01	0.13	0.01
FruitU						1.00	0.32	0.14	0.02	-0.02	-0.11	-0.02	0.00	-0.14	0.05
FruitC							1.00	0.16	0.02	0.00	-0.03	0.06	0.07	-0.19	-0.03
VegC								1.00	0.05	-0.01	0.00	0.09	0.01	-0.05	-0.03
FishC									1.00	0.01	0.02	0.02	-0.01	0.01	0.01
PoultC										1.00	0.10	0.02	0.02	-0.01	0.02
MeatC											1.00	0.19	0.07	0.04	-0.02
EggC												1.00	0.09	0.02	-0.01
DairyC													1.00	-0.01	-0.01
Smok														1.00	0.02
Activ															1.00

Supplementary Table 3. Description of the Scottish postcodes and council areas of the individuals in the study.

Abbv.	Area	Nind	Height	Weight	BIA Fat	Waist	Hips	WHR	BMI	ABSI	Creat	TC	HDL	Postcodes included
AC	Aberdeen City	657	657	655	650	655	651	651	655	653	629	626	629	AB10, AB11, AB12, AB13, AB14, AB15, AB16, AB21, AB22, AB23, AB24, AB25, AB31, AB32
AS	Aberdeenshire	538	533	532	518	522	517	516	531	519	520	522	522	AB30, AB33, AB34, AB36, AB39, AB41, AB42, AB43, AB45, AB51, AB52, AB53, AB54, AB55, AB56, DD10, DD9
Ag	Angus	2655	2638	2628	2572	2578	2559	2550	2624	2557	2583	2589	2588	DD11, DD2, DD3, DD4, DD5, DD7, DD8
AB	Argyll & Bute	62	62	61	59	61	61	61	61	61	57	56	56	G84, PA20, PA21, PA23, PA27, PA28, PA30, PA31, PA32, PA34, PA35, PA37, PA42, PA61, PA67
C	Clackmannanshire	25	25	25	25	25	25	25	25	25	22	22	22	FK10, FK11, FK12, FK13, FK14
DG	Dumfries & Galloway	23	23	23	23	23	23	23	23	23	23	23	22	DG1, DG10, DG11, DG12, DG14, DG2, DG3, DG4, DG5, DG6, DG7, DG9
DC	Dundee City	64	63	63	62	61	60	60	63	61	62	62	62	DD1
EA	East Ayrshire	35	35	34	34	34	34	34	34	34	35	35	35	KA1, KA16, KA17, KA18, KA2, KA3, KA4, KA5
ED	East Dunbartonshire	872	870	871	853	866	865	865	870	863	834	836	834	G61, G62, G64, G66
EL	East Lothian	31	31	31	31	31	31	30	31	31	28	28	28	EH21, EH22, EH31, EH32, EH33, EH34, EH39, EH41, EH42
ER	East Renfrewshire	378	377	377	368	377	377	376	377	377	363	361	363	G46, G76, G77, G78
EC	Edinburgh City	244	244	243	238	243	244	243	243	241	234	234	233	EH1, EH10, EH11, EH12, EH13, EH14, EH15, EH16, EH17, EH18, EH2, EH27, EH29, EH3, EH30, EH4, EH5, EH52, EH6, EH7, EH8, EH9
Fa	Falkirk	62	61	62	62	61	61	61	61	60	58	57	57	EH49, EH51, FK1, FK2, FK4, FK5, FK6
Fi	Fife	291	290	290	284	283	283	282	290	283	280	282	282	DD6, KY1, KY10, KY11, KY12, KY14, KY15, KY16, KY2, KY3, KY4, KY5, KY6, KY7, KY8, KY9
GC	Glasgow City	2032	2030	2026	1990	2021	2017	2014	2026	2017	1936	1934	1927	G1, G11, G12, G13, G14, G15, G2, G20, G21, G22, G23, G3, G31, G32, G33, G34, G4, G40, G41, G42, G43, G44, G45, G5, G51, G52, G53
H	Highland	63	63	63	63	62	62	62	63	62	63	63	63	AB37, IV1, IV10, IV15, IV16, IV17, IV19, IV2, IV21, IV24, IV27, IV28, IV3, IV5, IV51, IV55, IV6, IV63, IV7, KW1, KW14, PH20, PH21, PH22, PH25, PH26, PH33, PH40
I	Inverclyde	35	35	35	34	35	35	35	35	35	34	34	34	PA13, PA14, PA15, PA16, PA18, PA19
ML	Midlothian	9	9	9	9	9	9	9	9	9	9	9	9	EH19, EH20, EH23, EH25, EH26, EH46
Mo	Moray	17	17	17	17	17	17	17	17	17	17	17	17	IV30, IV31, IV32, IV36
NA	North Ayrshire	66	66	65	62	65	66	65	65	64	61	59	59	KA11, KA12, KA13, KA15, KA20, KA21, KA22, KA23, KA25, KA27, KA28, KA30, PA17
NL	North Lanarkshire	198	197	197	192	198	198	198	197	197	193	192	192	EH47, EH48, G65, G67, G68, G69, G71, ML1, ML2, ML4, ML5, ML6, ML7
OI	Orkney Islands	1	1	1	1	1	1	1	1	1	1	1	1	KW15
PK	Perth & Kinross	1801	1794	1791	1763	1772	1769	1764	1788	1764	1753	1753	1752	KY13, PH1, PH10, PH11, PH12, PH13, PH14, PH15, PH16, PH18, PH2, PH3, PH4, PH5, PH6, PH7, PH8
R	Renfrewshire	302	302	302	297	301	302	301	302	301	283	282	280	PA1, PA10, PA11, PA12, PA2, PA3, PA4, PA5, PA6, PA7, PA8
SB	Scottish Borders	29	29	29	29	28	28	28	29	28	29	29	29	EH43, EH45, TD1, TD11, TD12, TD14, TD15, TD2, TD5, TD6, TD8
SI	Shetland Islands	8	8	8	8	8	8	8	8	8	8	8	8	ZE1, ZE2, ZE3
SA	South Ayrshire	57	57	57	56	57	57	57	57	57	54	52	52	KA10, KA19, KA26, KA6, KA7, KA8, KA9
SL	South Lanarkshire	316	316	314	309	316	315	315	314	314	294	294	294	G72, G73, G74, G75, ML10, ML11, ML12, ML3, ML8, ML9
S	Stirling	73	73	73	72	73	73	73	73	73	72	72	72	FK15, FK16, FK17, FK19, FK20, FK7, FK8, FK9, G63
WD	West Dunbartonshire	150	150	150	147	150	149	149	150	150	137	136	136	G60, G81, G82, G83
WI	Western Isles	5	5	5	5	5	5	5	5	5	5	5	5	HS1, HS2, HS8
WL	West Lothian	19	19	19	19	19	19	19	19	19	19	19	19	EH53, EH54, EH55

Supplementary Table 4. Significance of region on phenotypes in the full framework. The values show the significance (p-values) of region in four models: 1. Differences in the traits (first column, Basal Model); 2. Correcting for kinship (second column, Family Model); 3. Correcting for kinship and genetic structure (third column, Structure Model); 4. Correcting for kinship and environmental covariates (fourth column, Environment Model); 5. Correcting for kinship and genetic and environmental covariates (fifth column, Structure and Environment Model). The asterisk marks the estimates that are significantly different from zero. All models corrected for sex, age and clinic.

	Model				
Trait	Basal	Family	Structure	Environment	Structure and Environment
Height	3.50E-06*	0.299	0.492	0.928	0.956
Weight	0.129	0.519	0.522	0.840	0.836
BIA Fat	0.002*	0.165	0.233	0.527	0.527
Waist	1.10E-05*	0.022*	0.042*	0.232	0.251
Hips	0.070	0.328	0.342	0.600	0.595
WHR	4.52E-07*	0.002*	0.009*	0.187	0.247
BMI	9.46E-06*	0.023*	0.040*	0.478	0.501
ABSI	2.70E-04*	0.006*	0.011*	0.033*	0.034*
Creatinine	1.81E-11*	1.15E-04*	1.51E-04*	1.62E-04*	2.03E-04*
TC	0.250	0.428	0.422	0.508	0.502
HDL	1.98E-05*	0.013*	0.016*	0.201	0.207

Supplementary Table 5. Variance explained by the fitted covariates in the model.

	Height	Weight	BIA Fat	Waist	Hips	WHR	BMI	ABSI	Creatinine	TC	HDL
SIMD	0.00120	0.00550	0.00410	0.00980	0.00670	0.00500	0.01280	0.00130		0.00080	0.00350
YE	0.00190	0.00110	0.00280	0.00260	0.00260	0.00130	0.00460				0.00140
Household Size	0.00100	0.00140		0.00080		0.00060	0.00050				
Vehicle Ratio	0.00040	0.00100					0.00060	0.00170		0.00320	
Jobless				0.00010	0.00010				0.00010		
Alcohol Units Std	0.00060	0.00040				0.00070			0.00230	0.00740	0.04760
Smoking Status									0.00130		
Active Status		0.11010	0.05770				0.11550	0.09370	0.16930		0.18880
Fruit Day Units	0.00030		0.00030					0.00120	0.00110	0.00200	0.00070
FruitC					0.02060			0.01270			
VeggiC	0.00090		0.00280			0.00120	0.00400	0.02620	0.01360		0.00640
FishC		0.00003	0.00010		0.00010	0.00010	0.00005	0.00010			0.00004
PoultC			0.00450	0.00590		0.01470		0.04640			0.01400
MeatC		0.00310	0.01240			0.00140	0.00710	0.03790	0.00280		0.00420
EggsC		0.01080	0.02400				0.01340	0.13210		0.01840	0.02070
DairyC			0.00020	0.00020	0.00040			0.00220	0.00020		0.00060
Total Environment	0.00640	0.13340	0.10890	0.01950	0.03040	0.02490	0.15850	0.35570	0.19060	0.03180	0.28800
gPC1	0.00320		0.00180	0.00150		0.00330	0.00150	0.00180			
gPC2	0.00030										
gPC3	0.00120								0.00040		
gPC4											
gPC5				0.00070		0.00040		0.00060			0.00120
gPC6											
gPC7											
gPC8											
gPC9											
gPC10											
Total gPCs	0.00480	0.00000	0.00180	0.00220	0.00000	0.00370	0.00150	0.00230	0.00040	0.00000	0.00120

Supplementary Figure 1. Changes in the standardised means for each trait per area before and after adjusting for the lifestyle and socioeconomic covariates. Yellow: regions with less than 20 individuals, not considered.

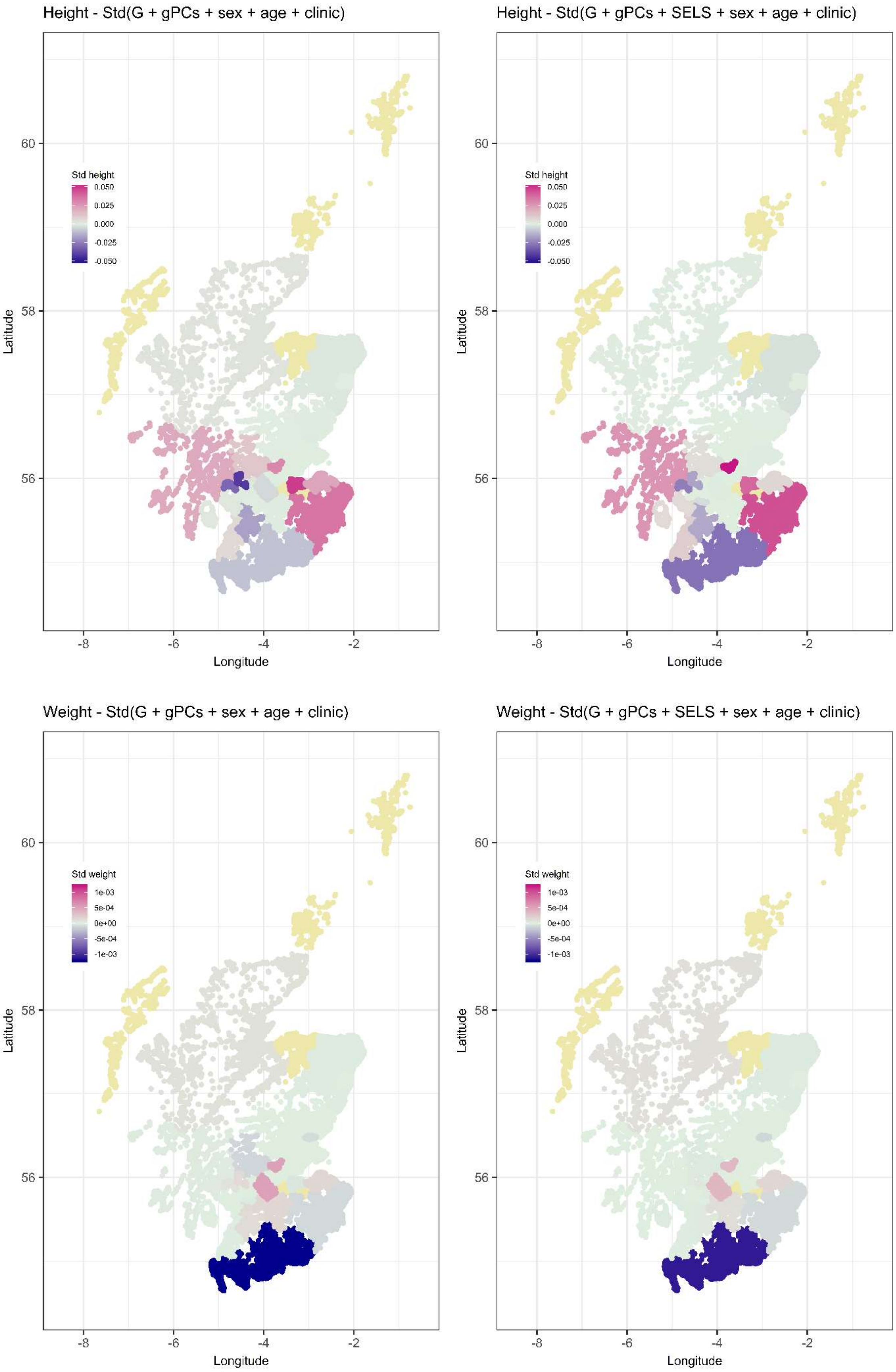

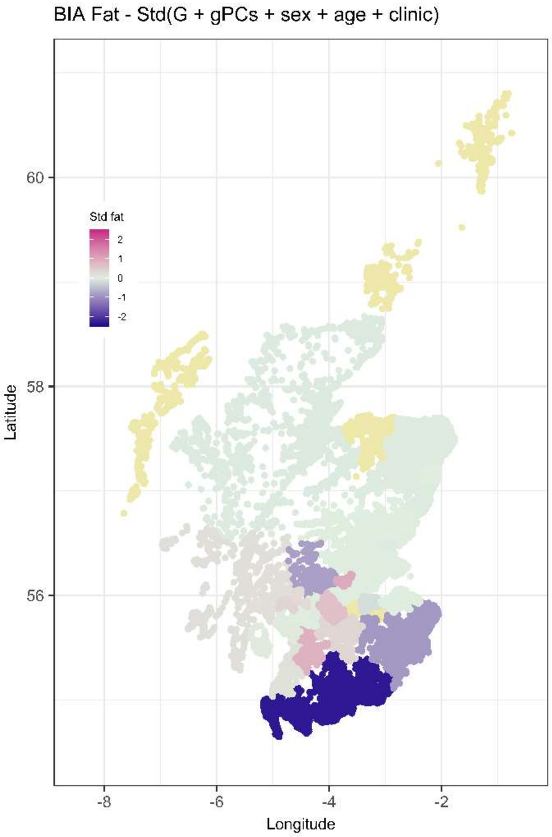
BIA Fat - Std(G + gPCs + sex + age + clinic)
Std fat
2
1
0
-1
-2
60
58
56
Latitude
-8
-6
-4
-2
Longitude

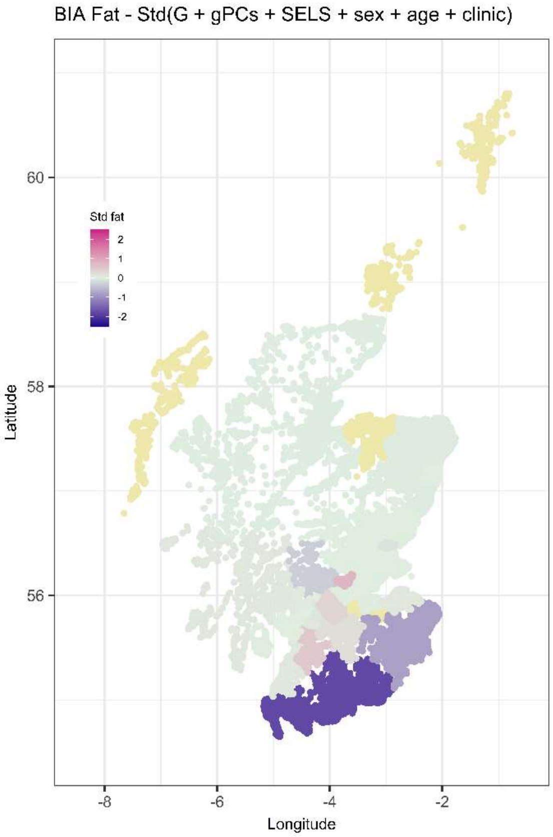
BIA Fat - Std(G + gPCs + SELS + sex + age + clinic)
Std fat
2
1
0
-1
-2
60
58
56
Latitude
-8
-6
-4
-2
Longitude

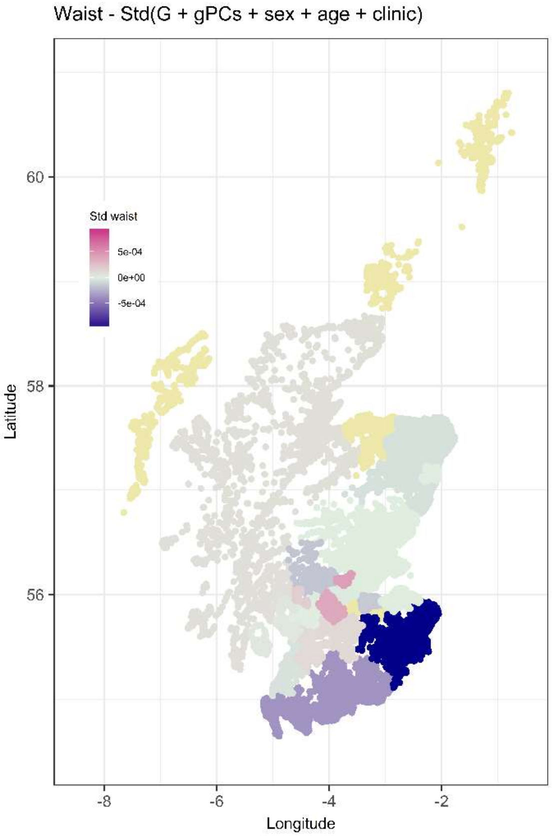
Waist - Std(G + gPCs + sex + age + clinic)
Std waist
5e-04
0e+00
-5e-04
60
58
56
Latitude
-8
-6
-4
-2
Longitude

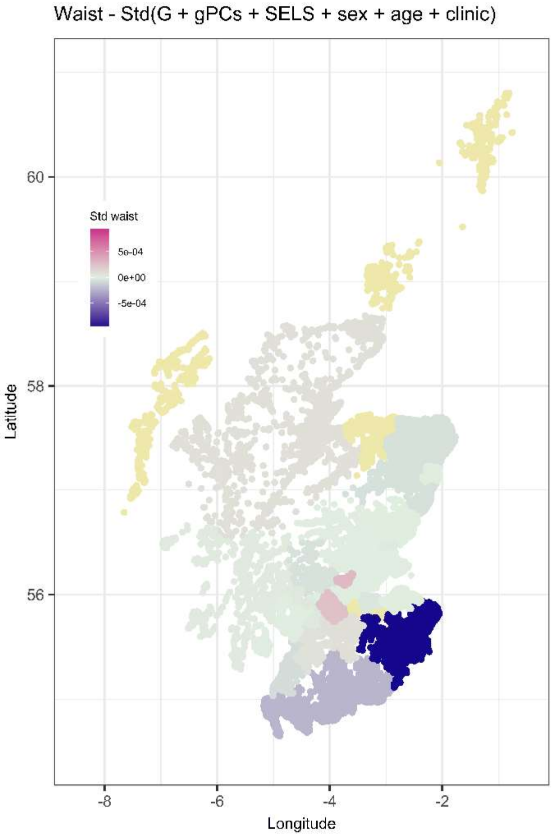
Waist - Std(G + gPCs + SELS + sex + age + clinic)
Std waist
5e-04
0e+00
-5e-04
60
58
56
Latitude
-8
-6
-4
-2
Longitude

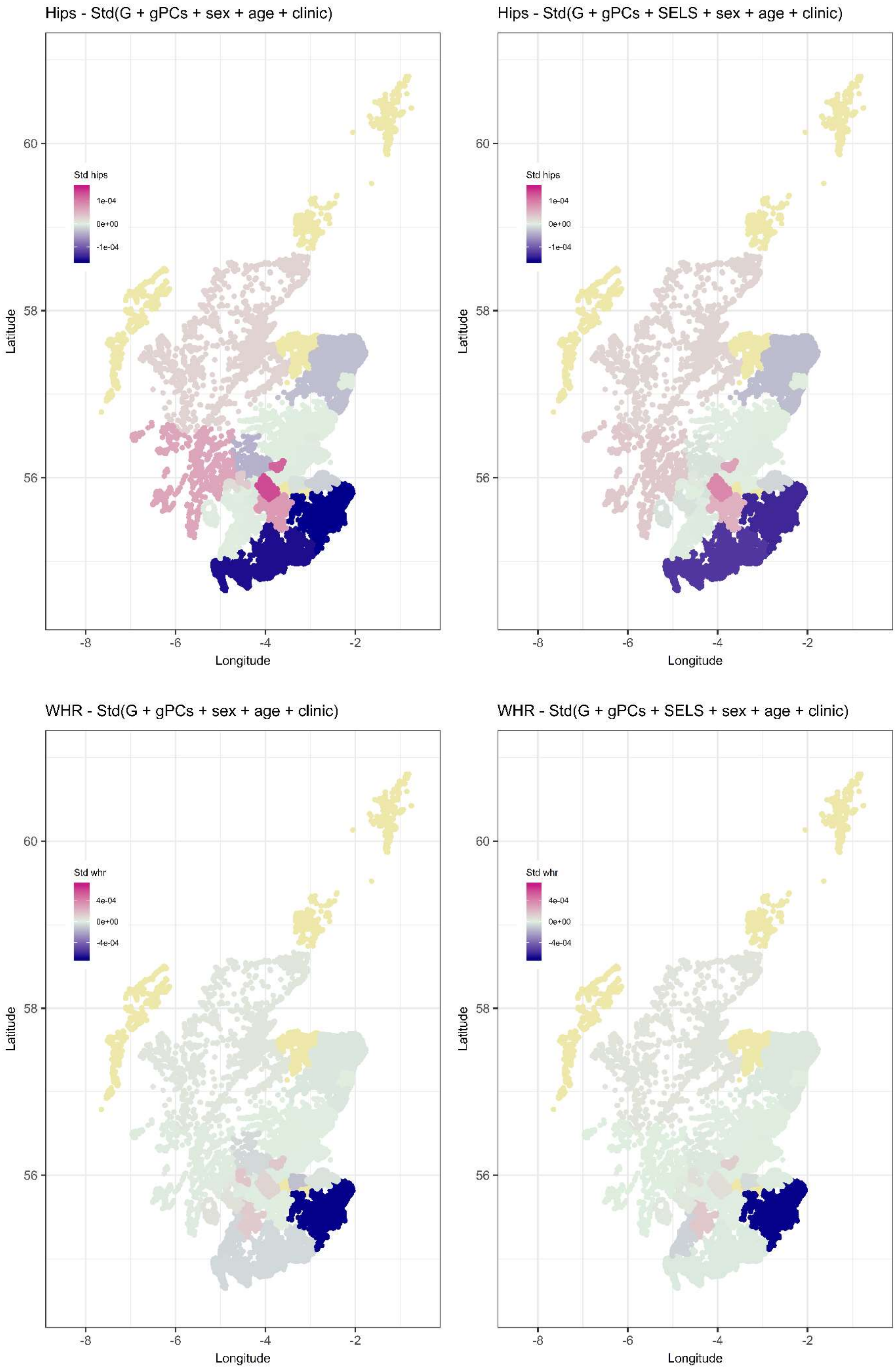
Hips - Std(G + gPCs + sex + age + clinic)
Std hips
1e-04
0e+00
-1e-04
Latitude
Longitude
Hips - Std(G + gPCs + SELS + sex + age + clinic)
WHR - Std(G + gPCs + sex + age + clinic)
Std whr
4e-04
0e+00
-4e-04
WHR - Std(G + gPCs + SELS + sex + age + clinic)

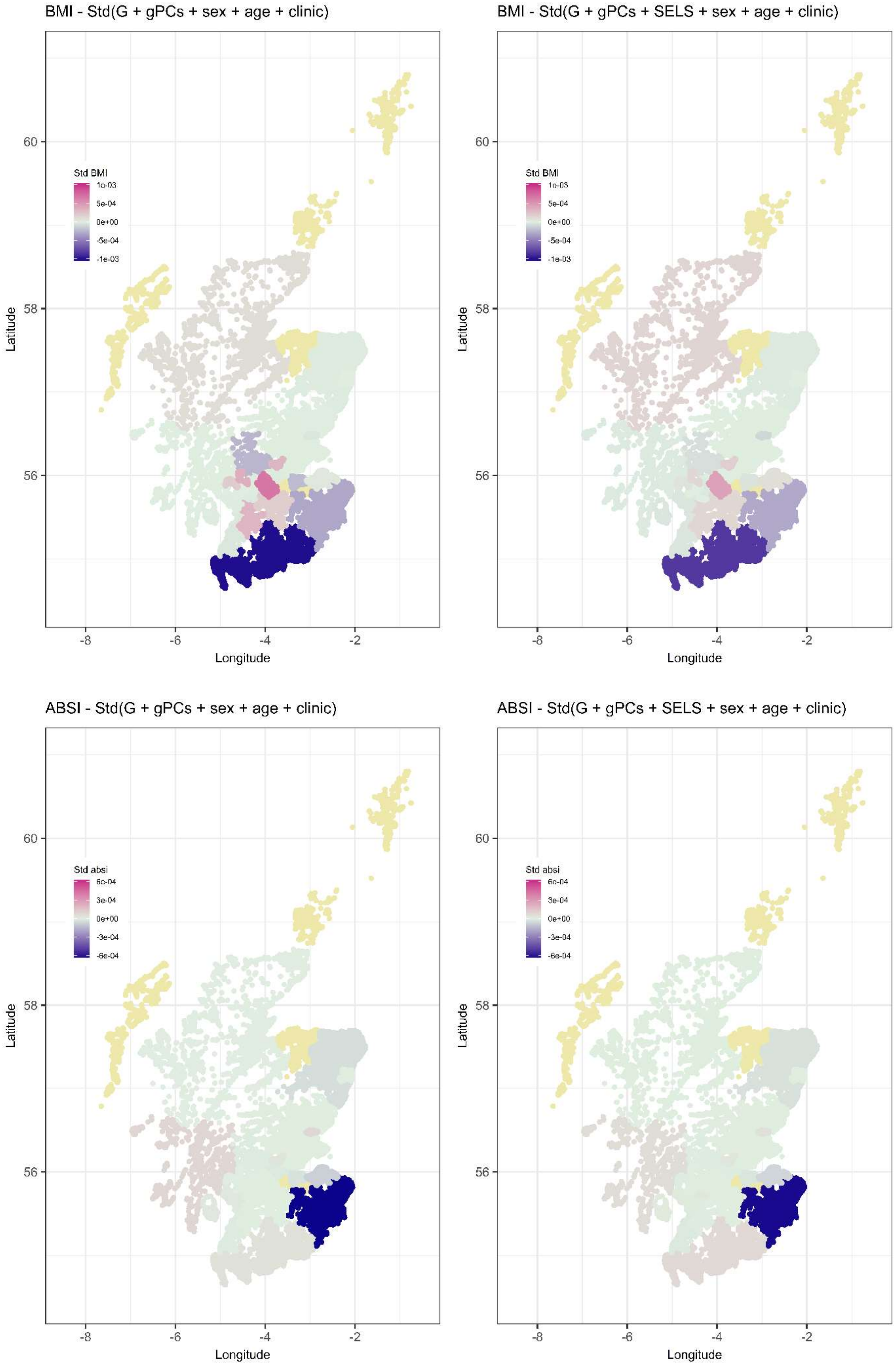
BMI - Std(G + gPCs + sex + age + clinic)
BMI - Std(G + gPCs + SELS + sex + age + clinic)
ABSI - Std(G + gPCs + sex + age + clinic)
ABSI - Std(G + gPCs + SELS + sex + age + clinic)
Std BMI
1e-03
5e-04
0e+00
-5e-04
-1e-03
Std absi
6e-04
3e-04
0e+00
-3e-04
-6e-04
Latitude
Longitude
60
58
56
-8
-6
-4
-2

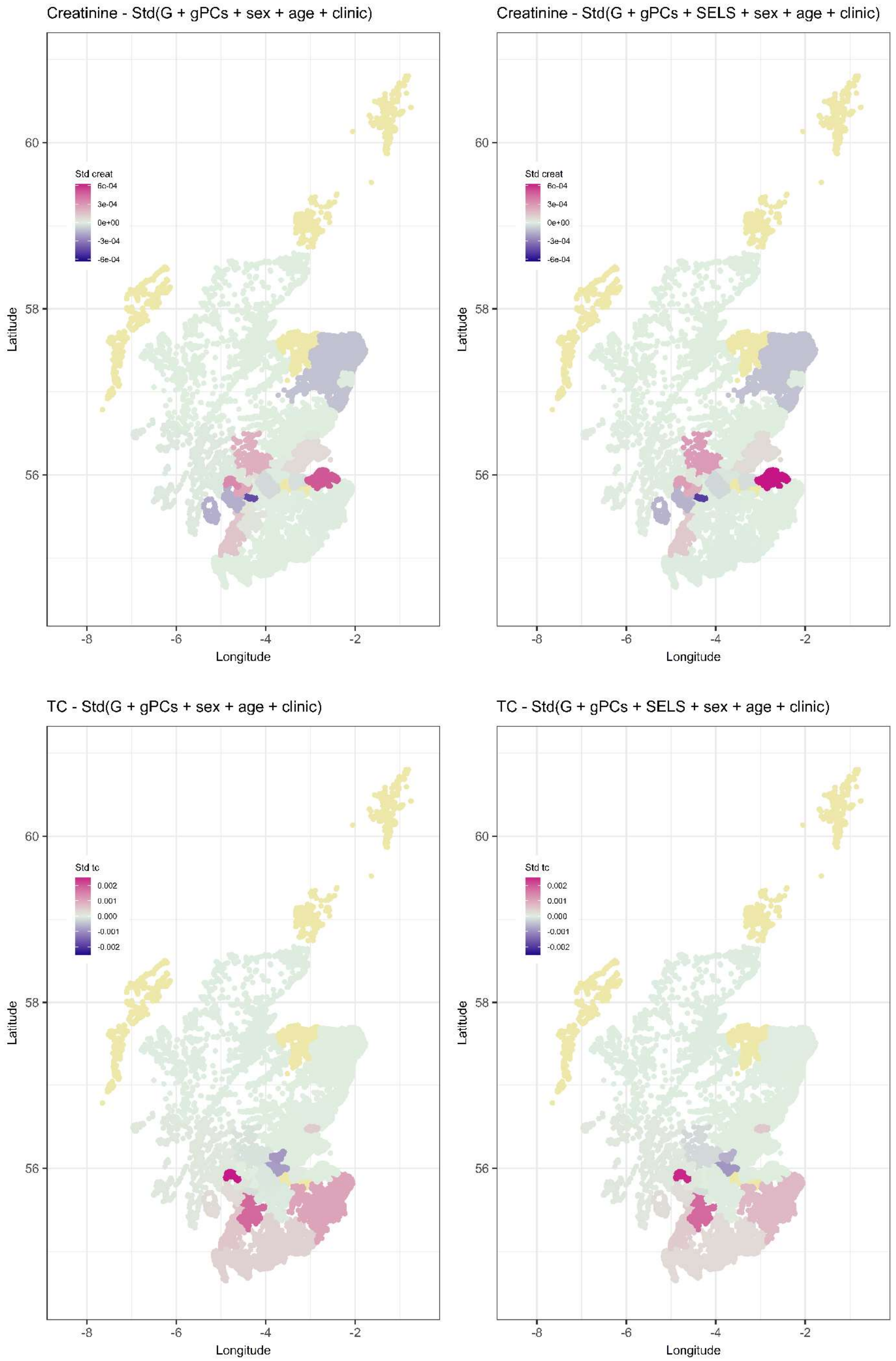
Creatinine - Std(G + gPCs + sex + age + clinic)
Creatinine - Std(G + gPCs + SELS + sex + age + clinic)
TC - Std(G + gPCs + sex + age + clinic)
TC - Std(G + gPCs + SELS + sex + age + clinic)
Std creat
6e-04
3e-04
0e+00
-3e-04
-6e-04
Std tc
0.002
0.001
0.000
-0.001
-0.002
Latitude
60
58
56
Longitude
-8
-6
-4
-2

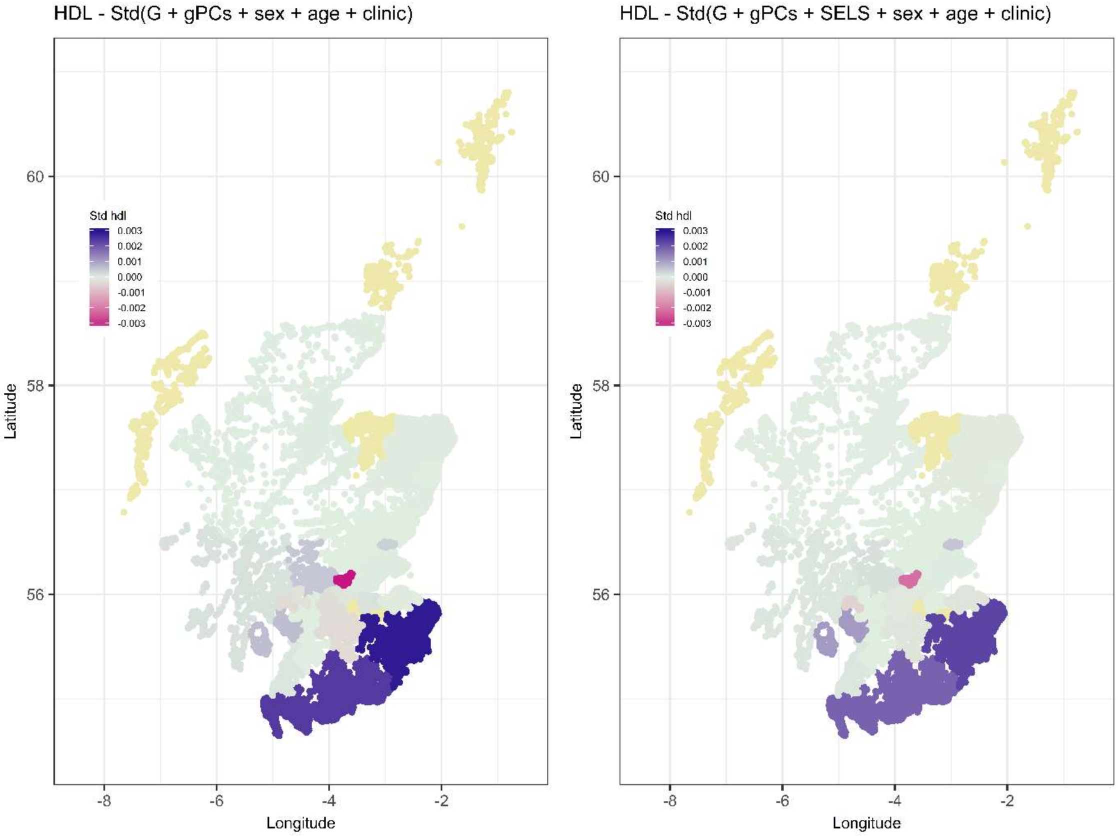
HDL - Std(G + gPCs + sex + age + clinic)
HDL - Std(G + gPCs + SELS + sex + age + clinic)
Std hdl
0.003
0.002
0.001
0.000
-0.001
-0.002
-0.003
Latitude
60
58
56
Longitude
-8
-6
-4
-2

Supplementary Figure 2. Boxplot of the traits in the different council areas.

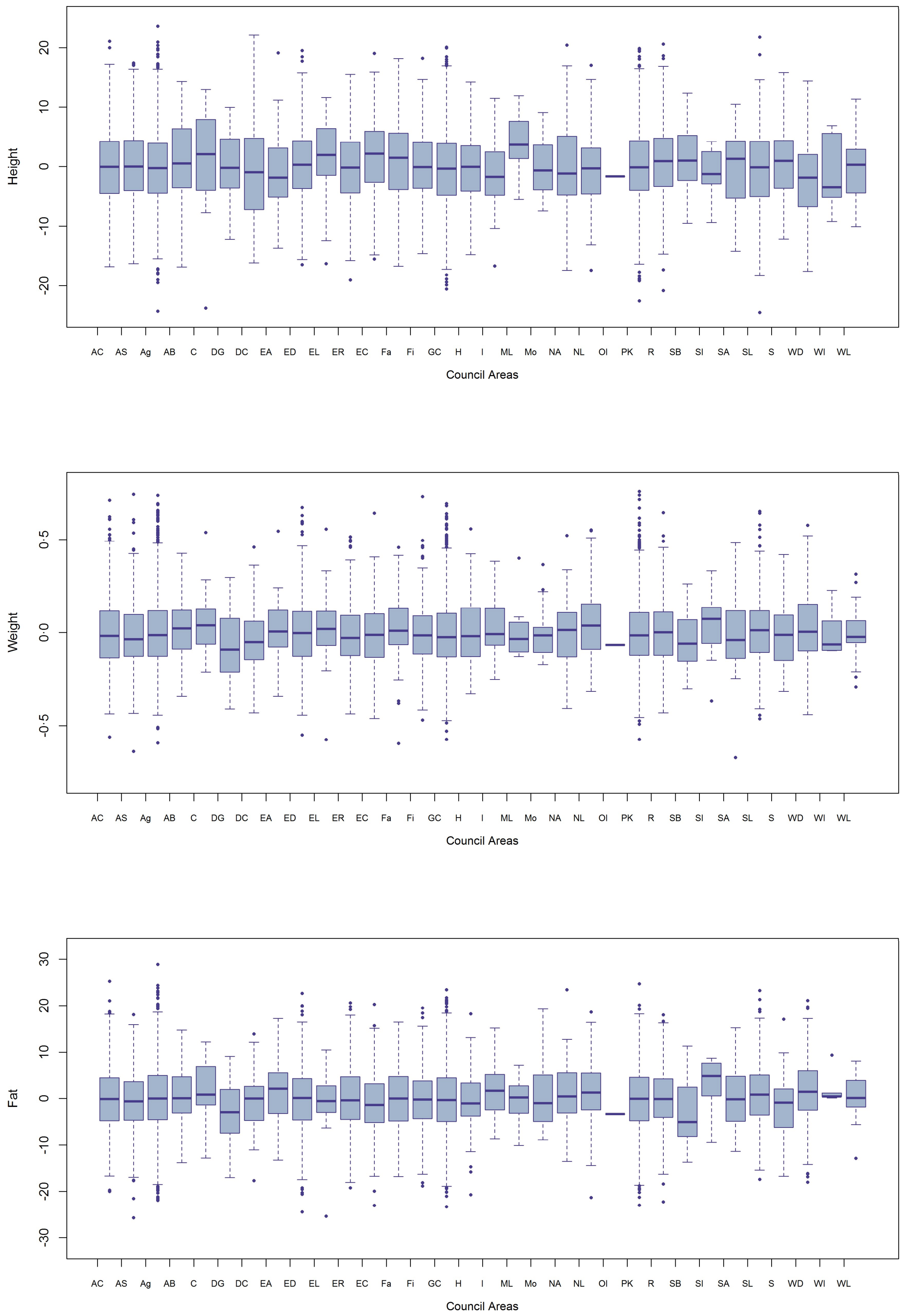

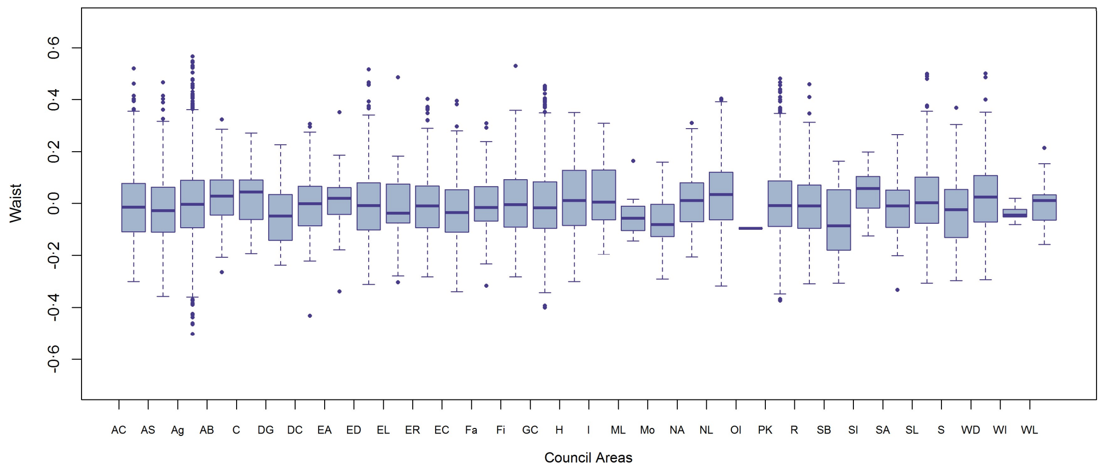
Waist
0·6
0·4
0·2
0·0
-0·2
-0·4
-0·6
AC AS Ag AB C DG DC EA ED EL ER EC Fa Fi GC H I ML Mo NA NL OI PK R SB SI SA SL S WD WI WL
Council Areas

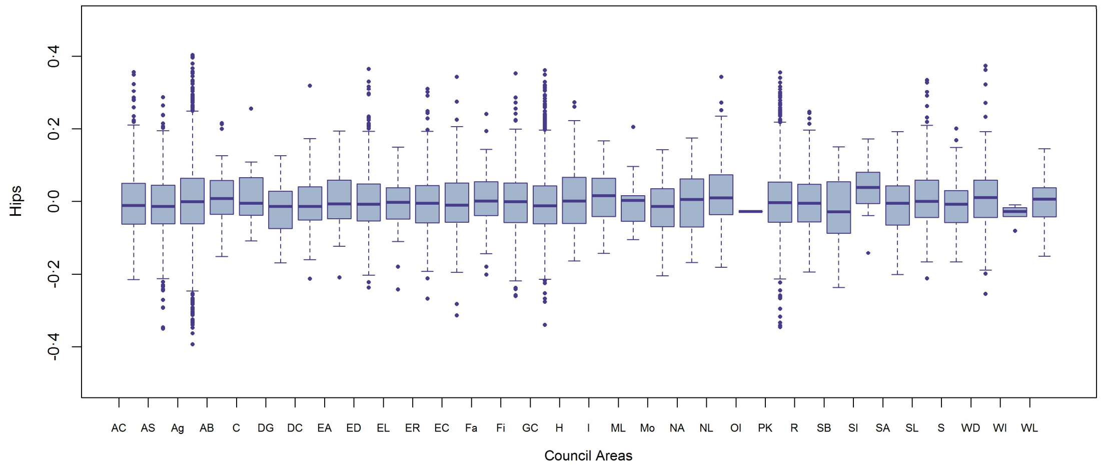
Hips
0·4
0·2
0·0
-0·2
-0·4
AC AS Ag AB C DG DC EA ED EL ER EC Fa Fi GC H I ML Mo NA NL OI PK R SB SI SA SL S WD WI WL
Council Areas

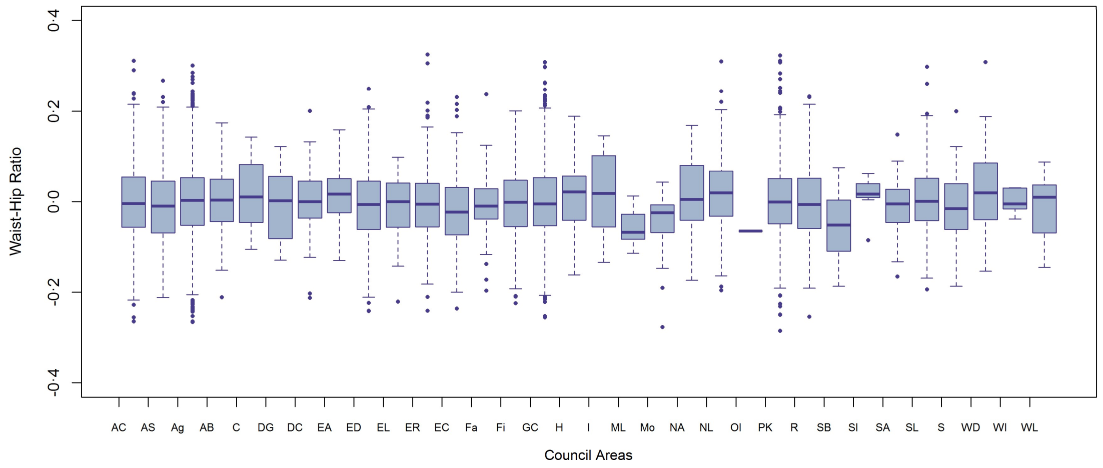
Waist-Hip Ratio
0·4
0·2
0·0
-0·2
-0·4
AC AS Ag AB C DG DC EA ED EL ER EC Fa Fi GC H I ML Mo NA NL OI PK R SB SI SA SL S WD WI WL
Council Areas

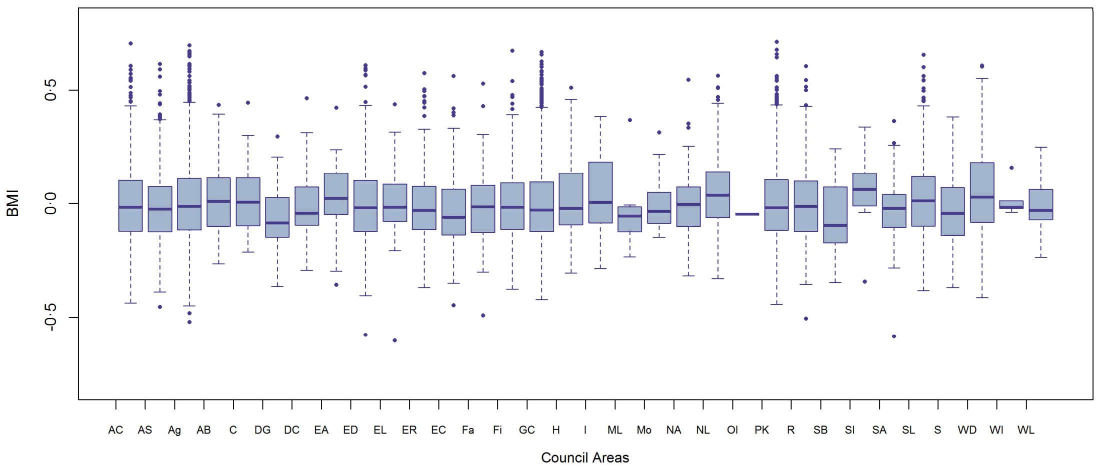
BMI
0·5
0·0
-0·5
AC AS Ag AB C DG DC EA ED EL ER EC Fa Fi GC H I ML Mo NA NL OI PK R SB SI SA SL S WD WI WL
Council Areas

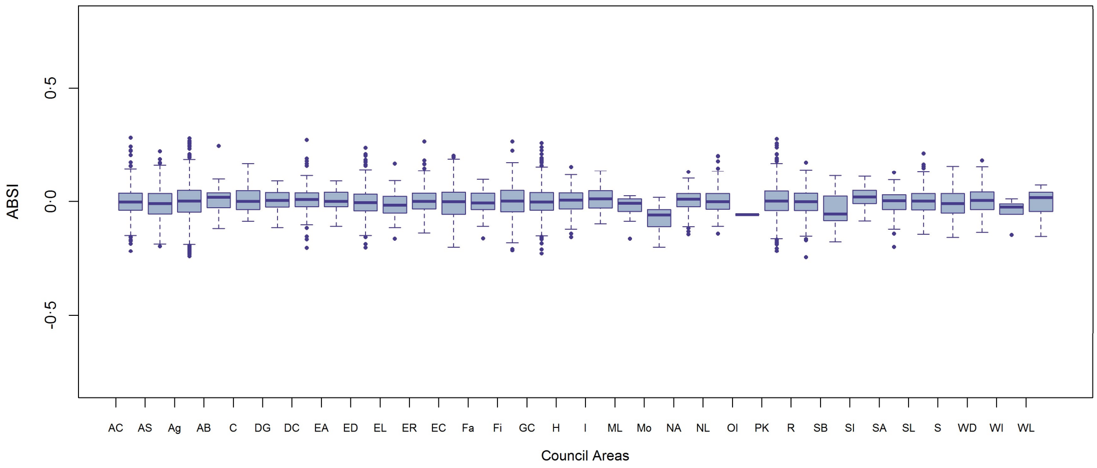
ABSI
0·5
0·0
-0·5
AC AS Ag AB C DG DC EA ED EL ER EC Fa Fi GC H I ML Mo NA NL OI PK R SB SI SA SL S WD WI WL
Council Areas

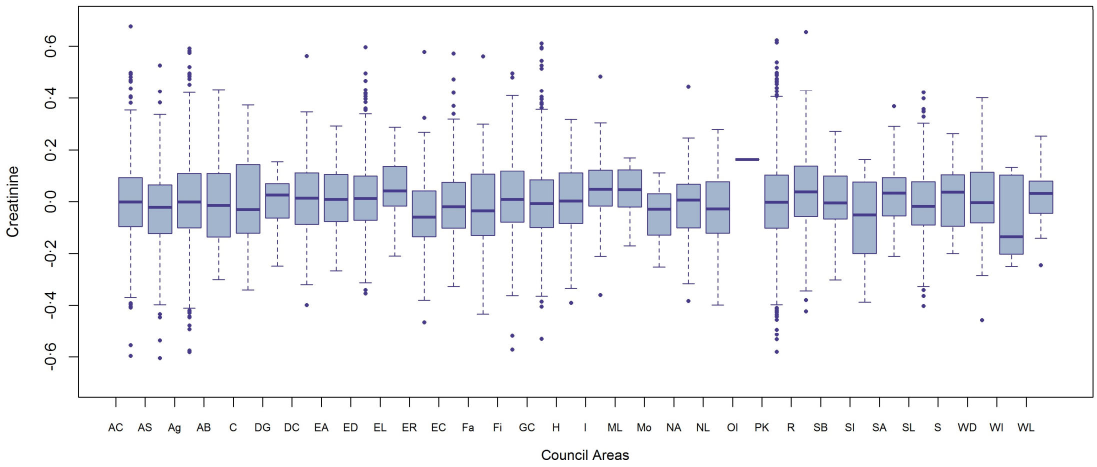
Creatinine
0·6
0·4
0·2
0·0
-0·2
-0·4
-0·6
AC AS Ag AB C DG DC EA ED EL ER EC Fa Fi GC H I ML Mo NA NL OI PK R SB SI SA SL S WD WI WL
Council Areas

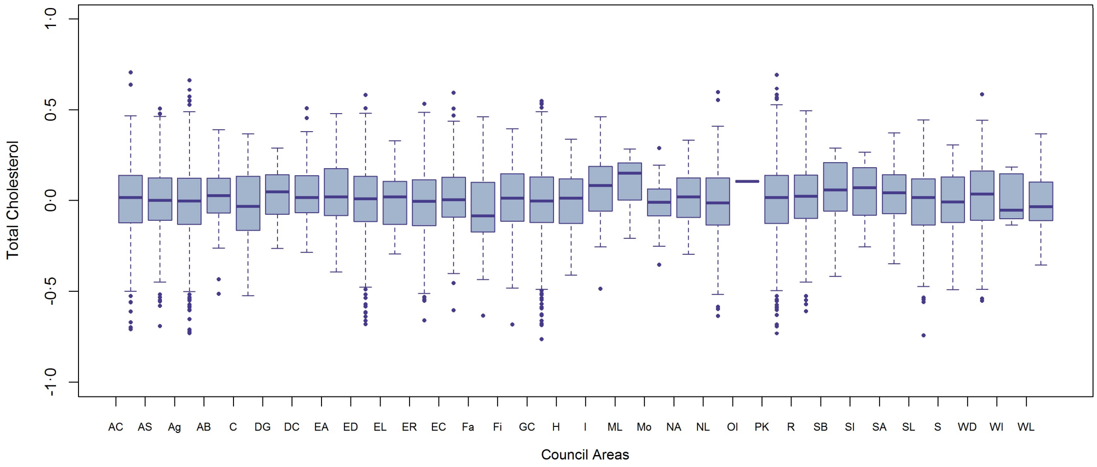

1·0
0·5
0·0
-0·5
-1·0
Total Cholesterol
AC AS Ag AB C DG DC EA ED EL ER EC Fa Fi GC H I ML Mo NA NL OI PK R SB SI SA SL S WD WI WL
Council Areas

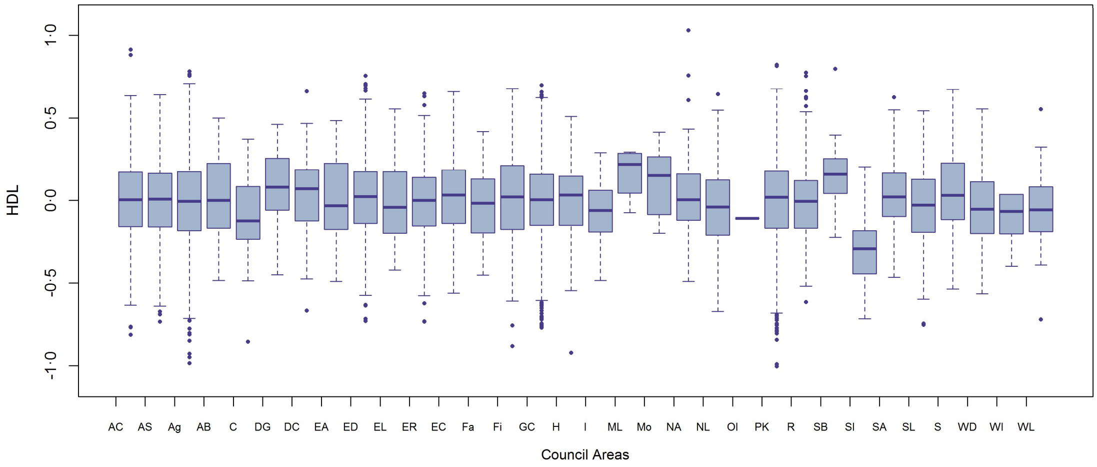

1·0
0·5
0·0
-0·5
-1·0
HDL
AC AS Ag AB C DG DC EA ED EL ER EC Fa Fi GC H I ML Mo NA NL OI PK R SB SI SA SL S WD WI WL
Council Areas

Supplementary Figure 3. Boxplot of the covariates in the different council areas.

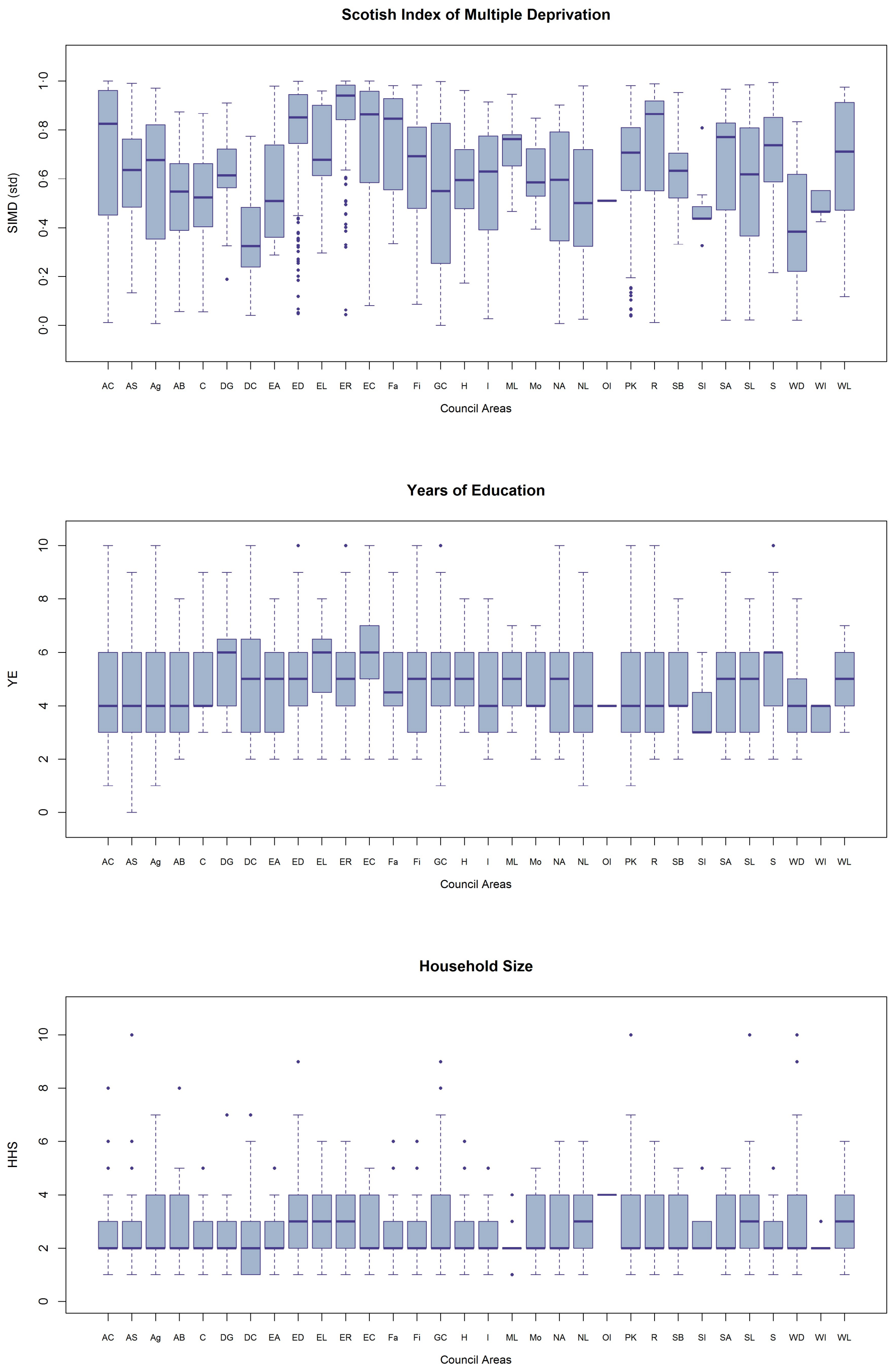

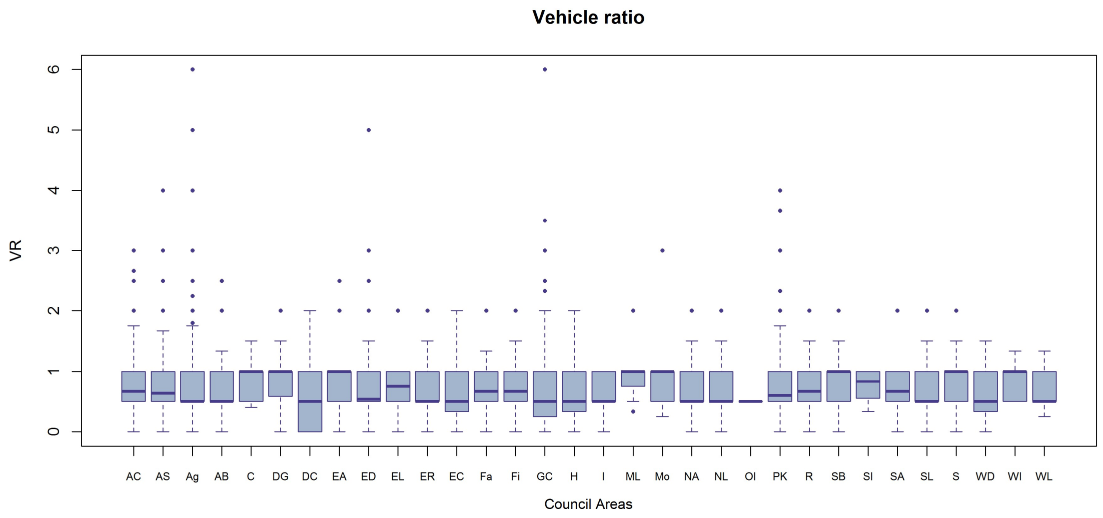
Vehicle ratio
VR
0
1
2
3
4
5
6
AC AS Ag AB C DG DC EA ED EL ER EC Fa Fi GC H I ML Mo NA NL OI PK R SB SI SA SL S WD WI WL
Council Areas

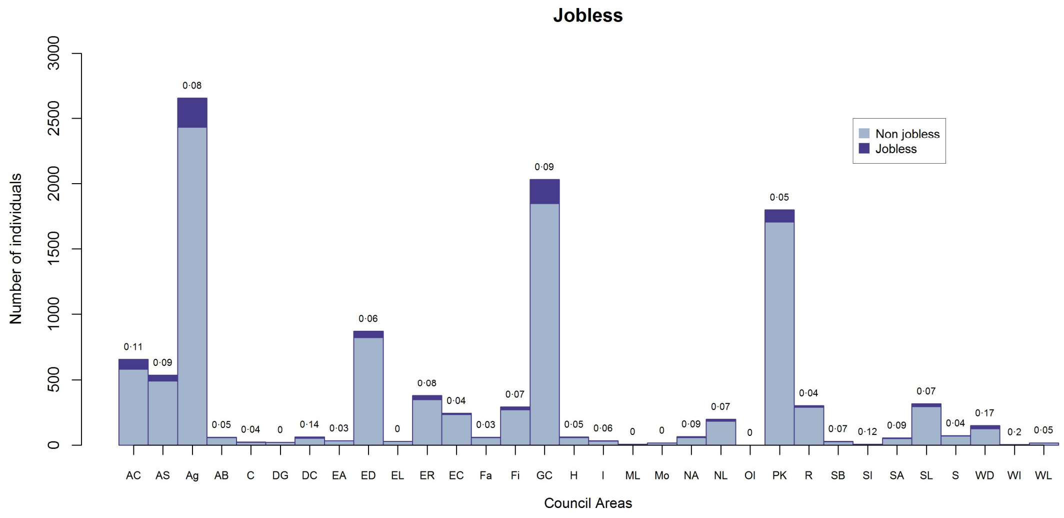
Jobless
Number of individuals
0
500
1000
1500
2000
2500
3000
Non jobless
Jobless
0·11 0·09 0·08 0·05 0·04 0 0·14 0·03 0·06 0 0·08 0·04 0·03 0·07 0·09 0·05 0·06 0 0 0·09 0·07 0 0·05 0·04 0·07 0·12 0·09 0·07 0·04 0·17 0·2 0·05
AC AS Ag AB C DG DC EA ED EL ER EC Fa Fi GC H I ML Mo NA NL OI PK R SB SI SA SL S WD WI WL
Council Areas

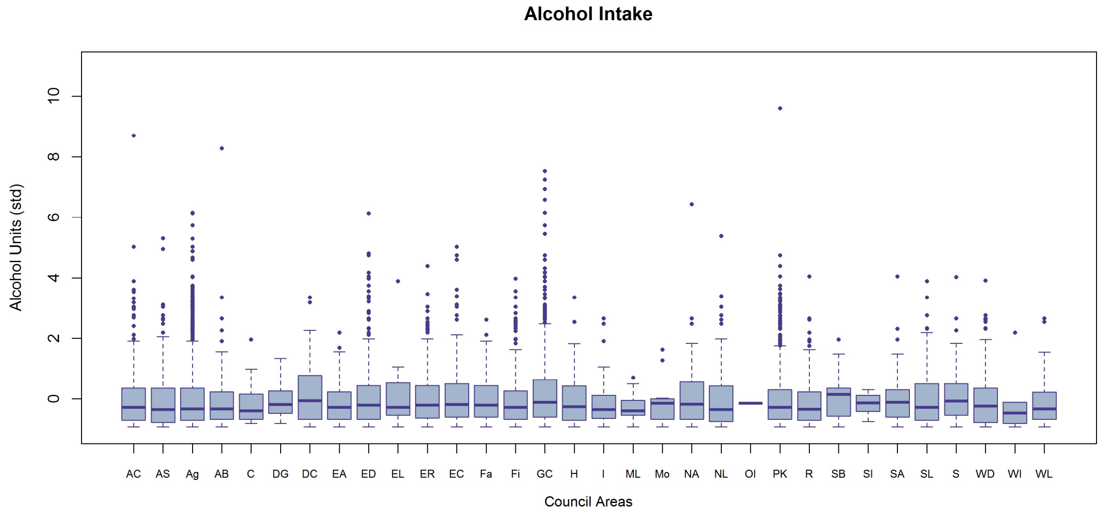
Alcohol Intake
Alcohol Units (std)
0
2
4
6
8
10
AC AS Ag AB C DG DC EA ED EL ER EC Fa Fi GC H I ML Mo NA NL OI PK R SB SI SA SL S WD WI WL
Council Areas

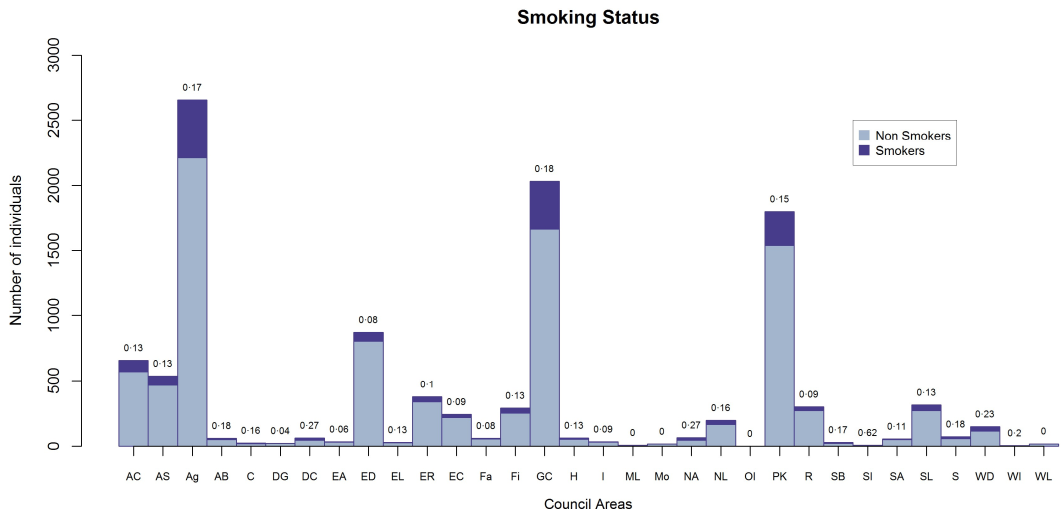
Smoking Status
Number of individuals
0 500 1000 1500 2000 2500 3000
Non Smokers
Smokers
0·13 0·13 0·17 0·18 0·16 0·04 0·27 0·06 0·08 0·13 0·1 0·09 0·08 0·13 0·18 0·13 0·09 0 0 0·27 0·16 0 0·15 0·09 0·17 0·62 0·11 0·13 0·18 0·23 0·2 0
AC AS Ag AB C DG DC EA ED EL ER EC Fa Fi GC H I ML Mo NA NL OI PK R SB SI SA SL S WD WI WL
Council Areas

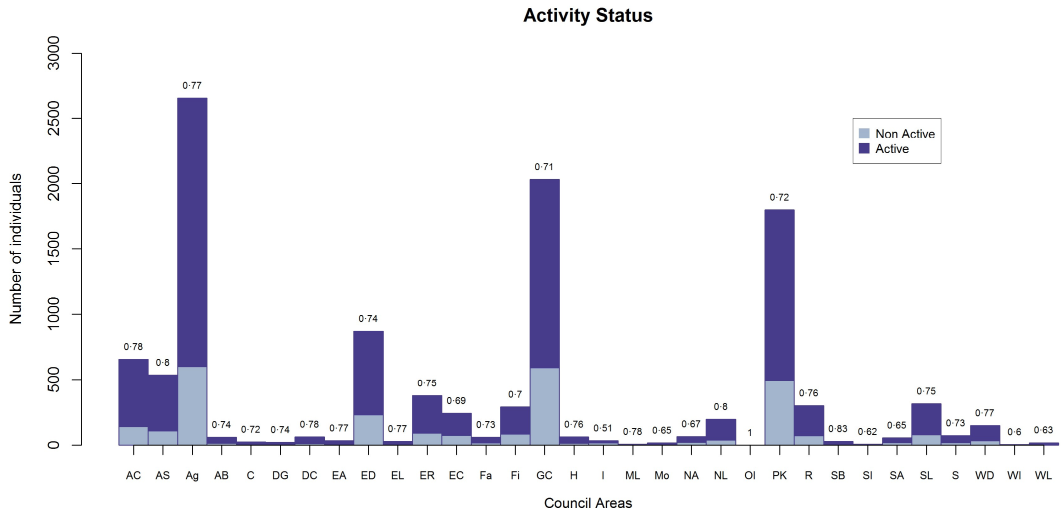
Activity Status
Number of individuals
0 500 1000 1500 2000 2500 3000
Non Active
Active
0·78 0·8 0·77 0·74 0·72 0·74 0·78 0·77 0·74 0·77 0·75 0·69 0·73 0·7 0·71 0·76 0·51 0·78 0·65 0·67 0·8 1 0·72 0·76 0·83 0·62 0·65 0·75 0·73 0·77 0·6 0·63
AC AS Ag AB C DG DC EA ED EL ER EC Fa Fi GC H I ML Mo NA NL OI PK R SB SI SA SL S WD WI WL
Council Areas

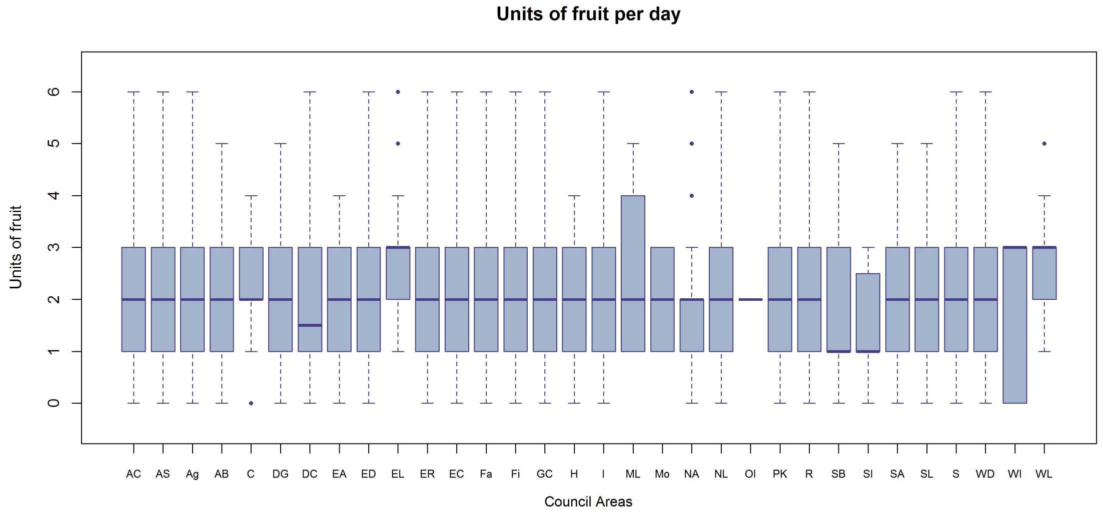
Units of fruit per day
Units of fruit
0 1 2 3 4 5 6
AC AS Ag AB C DG DC EA ED EL ER EC Fa Fi GC H I ML Mo NA NL OI PK R SB SI SA SL S WD WI WL
Council Areas

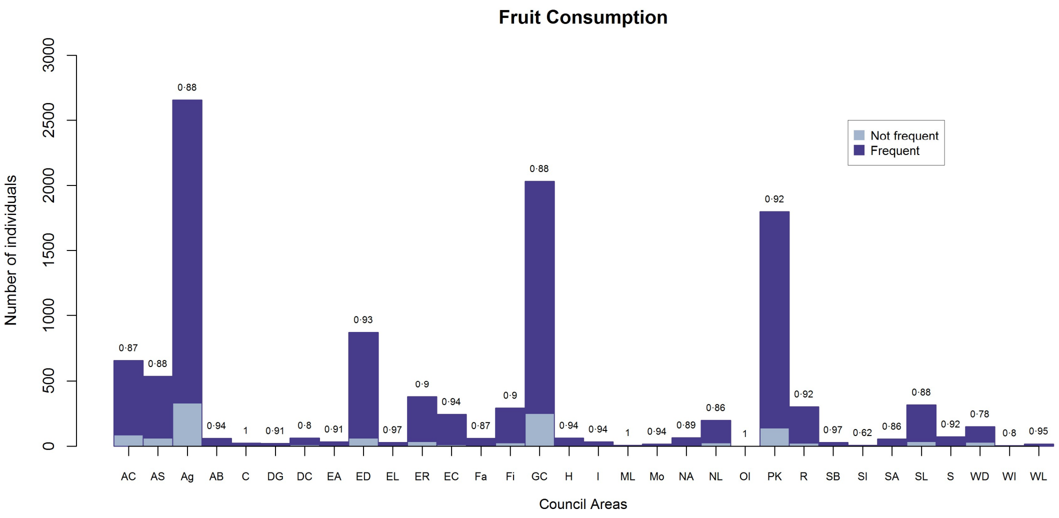
Fruit Consumption
Number of individuals
0
500
1000
1500
2000
2500
3000
Not frequent
Frequent
0·87 0·88 0·88 0·94 1 0·91 0·8 0·91 0·93 0·97 0·9 0·94 0·87 0·9 0·88 0·94 0·94 1 0·94 0·89 0·86 1 0·92 0·92 0·97 0·62 0·86 0·88 0·92 0·78 0·8 0·95
AC AS Ag AB C DG DC EA ED EL ER EC Fa Fi GC H I ML Mo NA NL OI PK R SB SI SA SL S WD WI WL
Council Areas

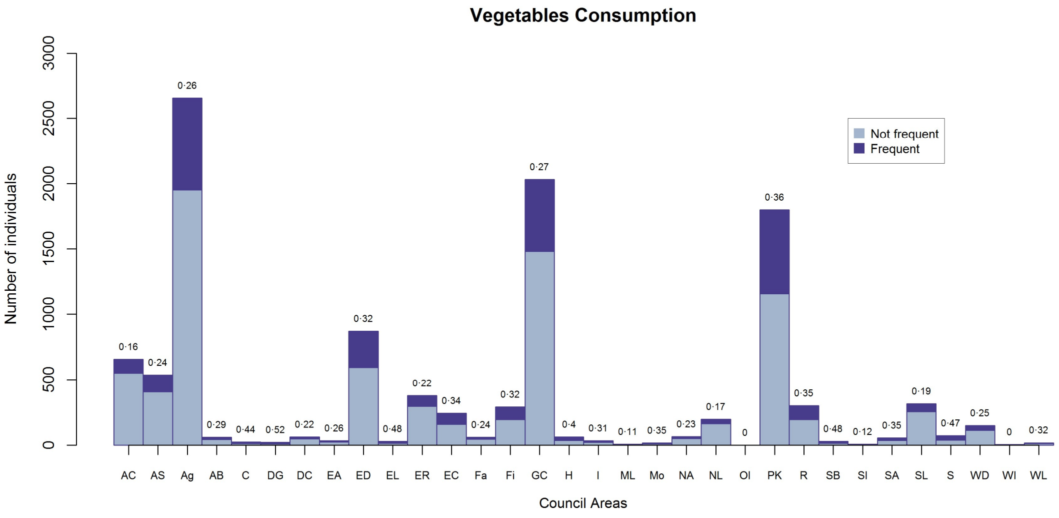
Vegetables Consumption
Number of individuals
0
500
1000
1500
2000
2500
3000
Not frequent
Frequent
0·16 0·24 0·26 0·29 0·44 0·52 0·22 0·26 0·32 0·48 0·22 0·34 0·24 0·32 0·27 0·4 0·31 0·11 0·35 0·23 0·17 0 0·36 0·35 0·48 0·12 0·35 0·19 0·47 0·25 0 0·32
AC AS Ag AB C DG DC EA ED EL ER EC Fa Fi GC H I ML Mo NA NL OI PK R SB SI SA SL S WD WI WL
Council Areas

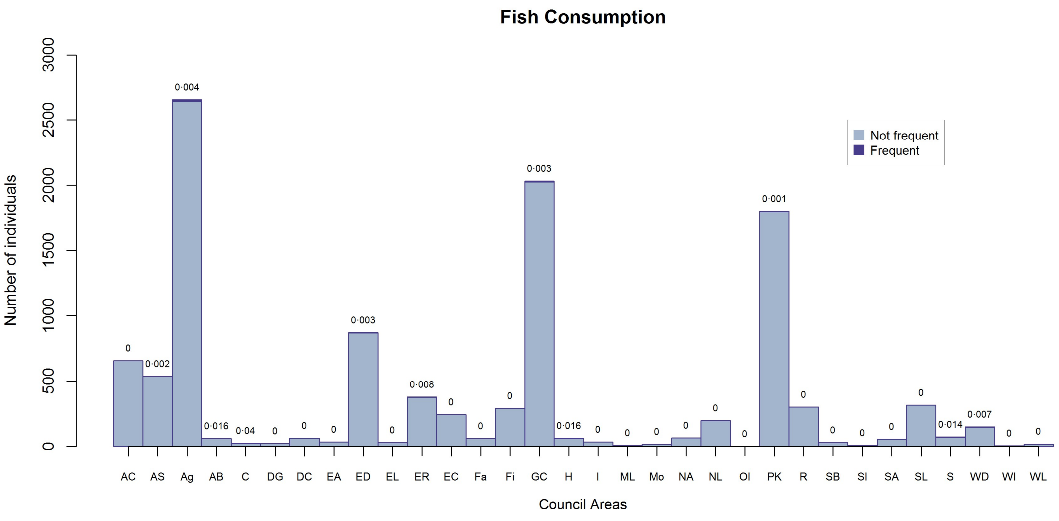
Fish Consumption
Number of individuals
0
500
1000
1500
2000
2500
3000
Not frequent
Frequent
0 0·002 0·004 0·016 0·04 0 0 0 0·003 0 0·008 0 0 0 0·003 0·016 0 0 0 0 0 0 0·001 0 0 0 0 0 0·014 0·007 0 0
AC AS Ag AB C DG DC EA ED EL ER EC Fa Fi GC H I ML Mo NA NL OI PK R SB SI SA SL S WD WI WL
Council Areas

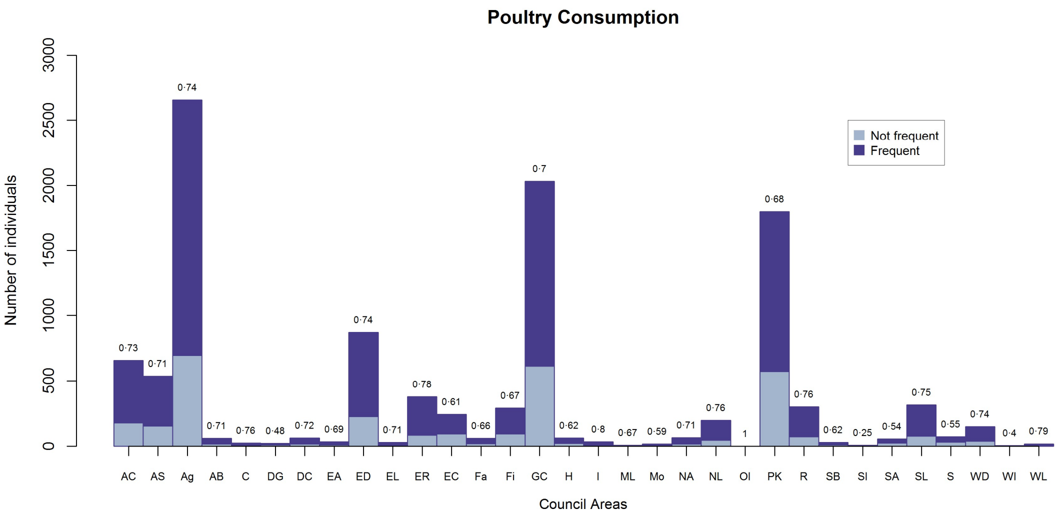
Poultry Consumption
Number of individuals
0 500 1000 1500 2000 2500 3000
Not frequent
Frequent
0·73 0·71 0·74 0·71 0·76 0·48 0·72 0·69 0·74 0·71 0·78 0·61 0·66 0·67 0·7 0·62 0·8 0·67 0·59 0·71 0·76 1 0·68 0·76 0·62 0·25 0·54 0·75 0·55 0·74 0·4 0·79
AC AS Ag AB C DG DC EA ED EL ER EC Fa Fi GC H I ML Mo NA NL OI PK R SB SI SA SL S WD WI WL
Council Areas

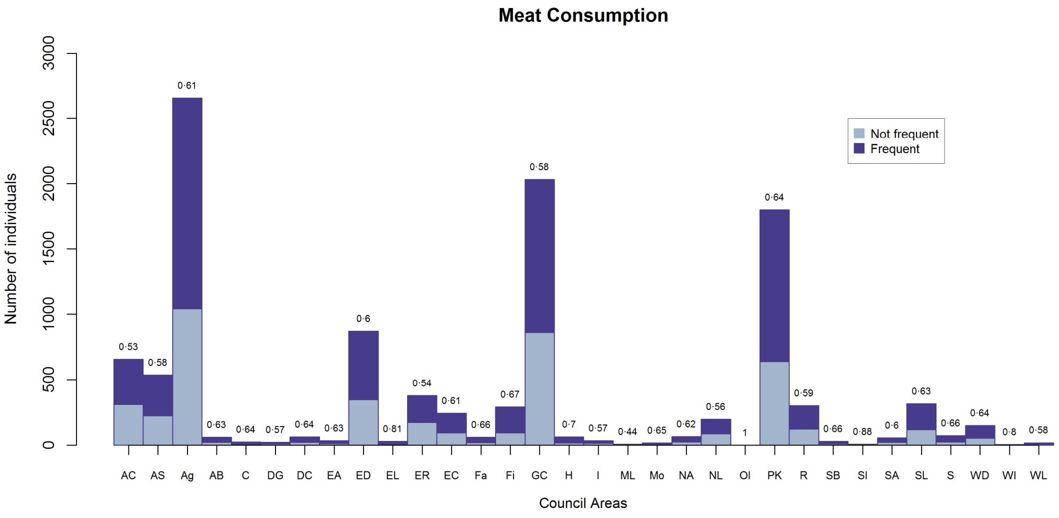
Meat Consumption
Number of individuals
0 500 1000 1500 2000 2500 3000
Not frequent
Frequent
0·53 0·58 0·61 0·63 0·64 0·57 0·64 0·63 0·6 0·81 0·54 0·61 0·66 0·67 0·58 0·7 0·57 0·44 0·65 0·62 0·56 1 0·64 0·59 0·66 0·88 0·6 0·63 0·66 0·64 0·8 0·58
AC AS Ag AB C DG DC EA ED EL ER EC Fa Fi GC H I ML Mo NA NL OI PK R SB SI SA SL S WD WI WL
Council Areas

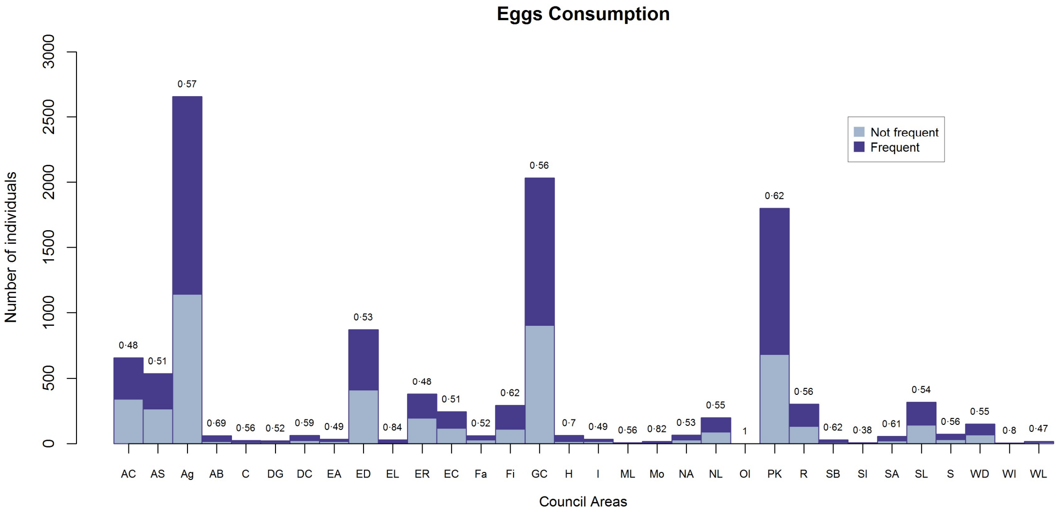
Eggs Consumption
Number of individuals
0 500 1000 1500 2000 2500 3000
Not frequent
Frequent
0·48 0·51 0·57 0·69 0·56 0·52 0·59 0·49 0·53 0·84 0·48 0·51 0·52 0·62 0·56 0·7 0·49 0·56 0·82 0·53 0·55 1 0·62 0·56 0·62 0·38 0·61 0·54 0·56 0·55 0·8 0·47
AC AS Ag AB C DG DC EA ED EL ER EC Fa Fi GC H I ML Mo NA NL OI PK R SB SI SA SL S WD WI WL
Council Areas

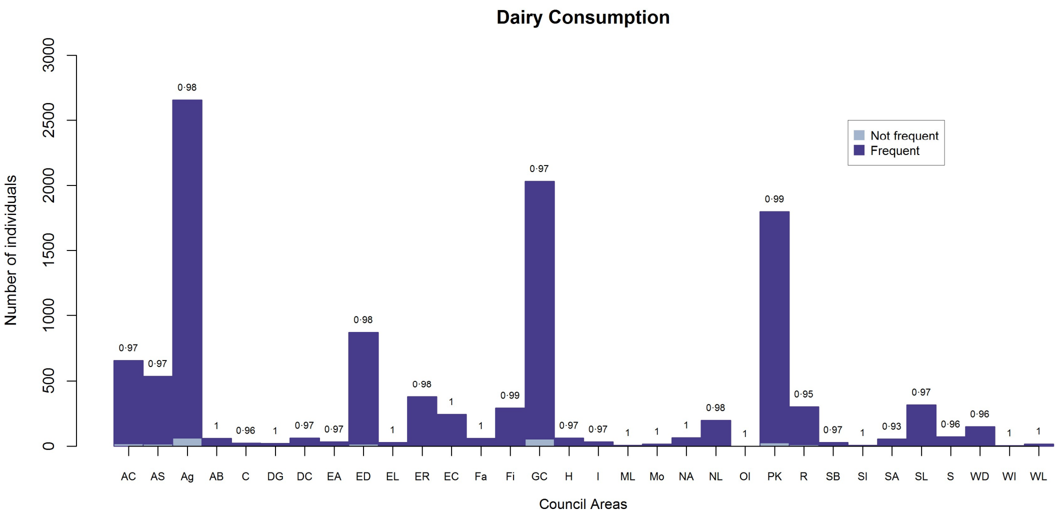

Dairy Consumption
Number of individuals
0
500
1000
1500
2000
2500
3000
Not frequent
Frequent
0·97
0·97
0·98
1
0·96
1
0·97
0·97
0·98
1
0·98
1
1
0·99
0·97
0·97
0·97
1
1
1
0·98
1
0·99
0·95
0·97
1
0·93
0·97
0·96
0·96
1
1
AC AS Ag AB C DG DC EA ED EL ER EC Fa Fi GC H I ML Mo NA NL OI PK R SB SI SA SL S WD WI WL
Council Areas

Supplementary Note 1. Quality control of lifestyle and socioeconomic variables.

Two different questionnaires (named V2 and V5) were taken by different subsets of the ~24,000 individuals in GS:SFHS. Each contained different questions to gather similar information. In order to obtain comparable values for variables collected from both questionnaires, so that information on each variable was available for all individuals, the quality control and standardisation described below was performed for each variable named.

<u>Alcohol units intake information</u>

In V2 people were asked number of units drank the previous week whereas in V5 they were asked about amount drank of specific drinks (also the previous week). V5 drinks were converted into units based on the values suggested in V2 for self-calculation of units, namely:

- 1 Glass of wine (red or white) = 1.5 units
- 1 Pint of beer/cider = 2 units
- 1 Bottle of beer/cider = 2 units
- 1 Measurement of spirits = 1 unit

After standardisation, data from both questionnaires were merged and an outlier removed (ID 128029 in V2, with more than 300 Units consumed during the week previous to answering the questionnaire).

<u>Smoking status information</u>

Both questionnaires shared the question *Have you ever smoked tobacco?* With possible answers:
1 - Yes, currently smoke; 2 - Yes, but stopped within past 12 months; 3 - Yes, but stopped more than 12 months ago; 4 - No, never smoked
The data was merged for that variable and the variable Smoking Status was created: 1: Smoker (4014 individuals), 0: NonSmoker (19265 individuals) (269 missing).

<u>Activity level information</u>

V2 separated work and leisure activities whereas V5 asked general questions and separated walking in a different category. To harmonise the information gathered by each questionnaire, 2 new measures summarising the hours per week spent doing vigorous and moderate activities were created (vigorous (VA) and moderate (MA) activity).
From V2, we obtained VA as the sum of time "very active" per week during work and during non-working time for vigorous activities; MA was the sum of time moderately active per week during work and during non-working time for moderate activity.
From V5, we obtained VA as the from the Time very active last week for vigorous activities and MA as the sum of time moderately active last week and the time spent walking last week for moderate activity.
The variables obtained were standardised before merging the data from the different questionnaires. For those individuals with missing values for VA but responses for MA, VA was set to zero instead to missing (assuming therefore that if they replied to moderate activity section and did not to vigorous activity one that they did not perform any vigorous activity).
The values for vigorous (VA) and moderate (MA) activity were converted into a new variable: Activity level, with 0 (corresponding to non-active) for those with VA_{std} <=0.5 and MA_{std} <=1, and 1 (active) for the rest.
17517 individuals were classified as active and 6031 as non-active.

<u>Diet information</u>

Individuals completing the V2 questionnaire were asked to quantify the amount of different foods eaten while those completing the V5 questionnaire were asked about the frequency of intake of the different types of food (possible answers were 1. Daily; 2. 5-6 times per week; 3. 2-4 times per week; 4. Once per week; 5. Less than once per week; 6. Less than once per month; 7. Never).
For responses to the V2 questionnaire, the amount consumed for each food group was converted into yearly consumption and that into the V5 intake frequency categories.
A new set of dichotomous variables was created from the categories. Intake for each food group was classified as "not usual consumption" (0, less than 1 day per week) or "usual consumption" (1, more than 1 day per week) creating the variables: Fruit Consumption, Vegetables Consumption (green vegetables + other

vegetables), Fish Consumption (oily and other types of fish), Poultry Consumption, Meat Consumption, Eggs Consumption and Dairy Consumption.
Since the calculation in V2 implied that the response could be a fractional number, when these occurred they were rounded to the closest integer. Individuals (always from V2, probably because of how the question was asked) with values >10 were considered outliers and set to missing.

A common variable for both questionnaires was Fruit Units (per day), so it was also merged.

Years of education
Both questionnaires shared the question *How many years altogether did you attend school/study full-time?*

The values are categorical but were fitted as a continuous variable in all the models. The meaning of the categories was:

0: 0; 1: 1-4;2: 5-9; 3: 10-11; 4: 12-13; 5: 14-15; 6: 16-17; 7: 18-19; 8: 20-21; 9: 22-23; 10: 24+
